# Massive expansion of human gut bacteriophage diversity

**DOI:** 10.1016/j.cell.2021.01.029

**Published:** 2021-02-18

**Authors:** Luis F. Camarillo-Guerrero, Alexandre Almeida, Guillermo Rangel-Pineros, Robert D. Finn, Trevor D. Lawley

**Affiliations:** 1 Host-Microbiota Interactions Laboratory, Wellcome Sanger Institute, Wellcome Genome Campus, Hinxton CB10 1SA, UK; 2 European Bioinformatics Institute (EMBL-EBI), Wellcome Genome Campus, Hinxton CB10 1SA, UK; 3 Wellcome Sanger Institute, Wellcome Genome Campus, Hinxton CB10 1SA, UK; 4 Max Planck Tandem Group in Computational Biology, Department of Biological Sciences, Universidad de los Andes, Bogota 111711, Colombia

**Keywords:** virus, phage, human gut, microbiome, database, metagenomics, gut bacteria

## Abstract

Bacteriophages drive evolutionary change in bacterial communities by creating gene flow networks that fuel ecological adaptions. However, the extent of viral diversity and its prevalence in the human gut remains largely unknown. Here, we introduce the Gut Phage Database, a collection of ∼142,000 non-redundant viral genomes (>10 kb) obtained by mining a dataset of 28,060 globally distributed human gut metagenomes and 2,898 reference genomes of cultured gut bacteria. Host assignment revealed that viral diversity is highest in the Firmicutes phyla and that ∼36% of viral clusters (VCs) are not restricted to a single species, creating gene flow networks across phylogenetically distinct bacterial species. Epidemiological analysis uncovered 280 globally distributed VCs found in at least 5 continents and a highly prevalent phage clade with features reminiscent of p-crAssphage. This high-quality, large-scale catalog of phage genomes will improve future virome studies and enable ecological and evolutionary analysis of human gut bacteriophages.

## Highlights

• Database containing 142,809 non-redundant gut phage genomes from 28,060 metagenomes
• Host assignment reveals phage diversity and host range across gut bacteria isolates
• Epidemiology analysis unveils 280 viral clusters that are globally distributed
• The Gubaphage is a clade that infects several members of the Bacteroidales order

## Graphical Abstract

**Figure:**
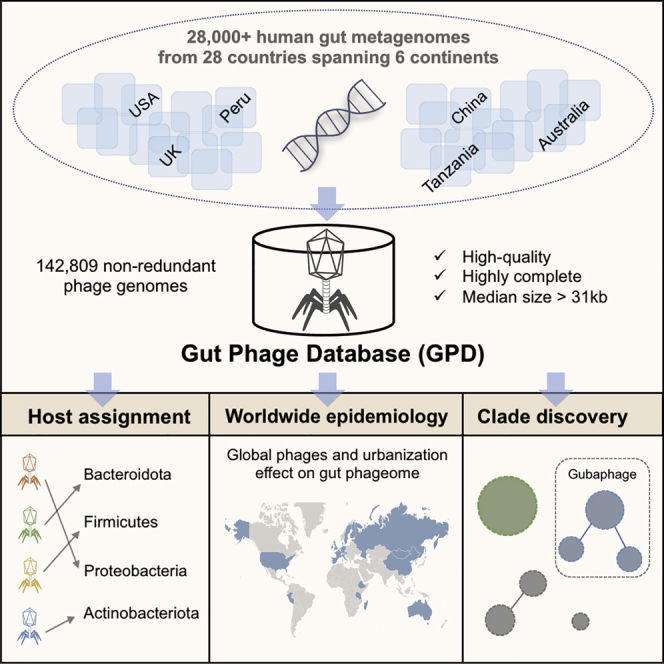

## Teaser

By mining human gut metagenomes and gut bacteria isolates, Camarillo-Guerrero et al. compile high-quality gut bacteriophage genomes into the Gut Phage Database (GPD) and analyze the diversity and worldwide distribution of phage.

## Introduction

Viruses are the most numerous biological entities on Earth with an estimated population size of 10^31^ particles (Brüssow and Hendrix, 2002). Bacteriophages (or phages; viruses that infect bacteria and archaea) profoundly influence microbial communities by functioning as vectors of horizontal gene transfer (Jain et al., 1999), encoding accessory functions of benefit to host bacterial species (Harrison and Brockhurst, 2017), and promoting dynamic co-evolutionary interactions (Betts et al., 2014). For decades, the discovery of phages occurred at a slow pace. However, with the advent of high-throughput metagenomics, it became possible to uncover an unparalleled amount of novel phage diversity (Al-Shayeb et al., 2020; Paez-Espino et al., 2016). A surprising finding was that the majority of phage sequences uncovered by metagenomics could not be classified into any known viral taxonomy laid out by the International Committee on Taxonomy of Viruses (ICTV) (e.g., species, genus, family) (Simmonds et al., 2017), prompting many researchers to organize phage predictions from metagenomic datasets into grouping schemes based solely on genomic features (Bin Jang et al., 2019).

The impact of phages on different ecosystems is beginning to be uncovered, with phages found in the oceans already being referred to as “puppet masters” due to their significant impact on oceanic biogeochemistry (Breitbart et al., 2018). Given the impact of the gut microbiome composition and function on human health, there is a growing focus on phages that inhabit the gut ecosystem (Clooney et al., 2019; Kho and Lal, 2018). The first metagenomic studies revealed that the majority (81%–93%) of the viral gut diversity is novel (Manrique et al., 2016; Reyes et al., 2010), but gut phage host assignment and host range remain largely uncharacterized. An exception has been p-crAssphage, a phage discovered in 2014 by computational analysis of metagenomic reads and found in >50% of western human gut microbiomes (Dutilh et al., 2014). Analyses of predicted phage sequences from gut metagenomes have yielded fascinating insights into phage biology, such as the presence of sticky domains (which could facilitate adherence of phage to the intestinal mucus; Barr et al., 2013), reverse transcriptases that promote gene hypervariation (Minot et al., 2012), and proteins with ankyrin domains that could aid bacterial hosts in immune evasion (Jahn et al., 2019).

Previous analyses have focused on bulk viral fragments with limited resolution to characterize individual phage genomes or link specific phages to a bacterial host species (Minot et al., 2012). More recently, human gut metagenomes have been mined to compile a more comprehensive list of gut phage genomes (Gregory et al., 2019; Paez-Espino et al., 2019), providing new fundamental insights into the viral diversity and functions present in the human gut microbiome. Nevertheless, the limited number (<700) of metagenomes used to construct these databases (GVD and gut phage fraction from IMG/VR), and the fragment size of their predictions (median size <15 kb as opposed to ∼50 kb for an average *Caudovirales* phage genome commonly found in the human gut), suggests that the majority of gut phage diversity remains uncharacterized and incomplete. Indeed, a recent report estimated that IMG/VR, which contains viral sequences from a wide range of environments, showed that only 1.9% of the predictions were complete, and 2.5% were classified as high quality (>90% complete) (Nayfach et al., 2020). A comprehensive resource of longer and complete reference phage genomes is required to enable genome-resolved metagenomics for gut phage studies across human populations.

Here, we introduce the Gut Phage Database (GPD), a highly curated database containing 142,809 non-redundant phage genomes derived from the analysis of 28,060 globally distributed metagenomic samples. Importantly, the GPD includes over 40,000 high-quality genomes with a median size of 47.68 kb. We use GPD to gain insight into the biology, host range, and global epidemiology of human gut phages. We uncover 280 globally distributed viral clusters, including 1 viral clade (Gubaphage) with reminiscent features to p-crAssphage. Given the high quality of the reference genomes, the database size, and the sequence diversity harbored by the GPD, this resource will greatly improve the characterization of individual human gut bacteriophages at a global or local scale.

## Results

### Generation of the GPD

**Figure 1:**
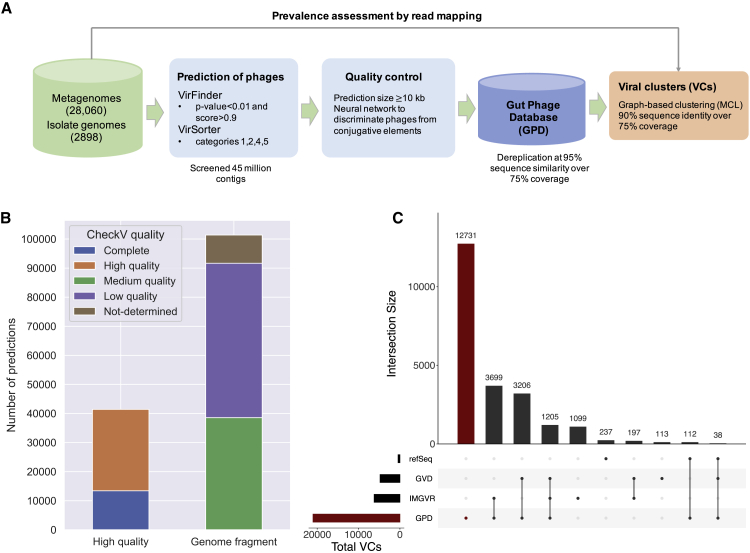
Generating the most complete sequence database of human gut bacteriophages (A) Massive prediction of phage genomes from 28,060 human gut metagenomes and 2,898 isolate genomes was carried out by using VirFinder and VirSorter with conservative settings. A machine learning approach (see STAR methods) was used to increase the quality of predictions and redundancy was removed by clustering the sequences at a 95% sequence identity. Diversity was further analyzed by generating VCs of predictions with a graph-based approach. (B) Quality estimation of GPD genomes by CheckV. Over 40,000 predictions are categorized as high-quality. (C) UpSet plot comparing GPD against other public gut phage databases. GPD captures the greatest unique diversity of phage genomes that inhabit the human gut.

In order to obtain a comprehensive view of human gut phage diversity, we analyzed 28,060 public human gut metagenomes and 2,898 bacterial isolate genomes cultured from the human gut (Figure 1A). To identify viral sequences among human gut metagenomes, we screened over 45 million assembled contigs with VirFinder (Ren et al., 2017), which relies on *k*-mer signatures to discriminate viral from bacterial contigs, and VirSorter (Roux et al., 2015), which exploits sequence similarity to known phage and other viral-like features such as guanine cytosine (GC) skew. Because obtaining high-quality genomes was essential for our downstream analyses, we used conservative settings (see STAR methods for further details) for both tools and retained only predictions that were at least 10 kb long.

**Figure S1:**
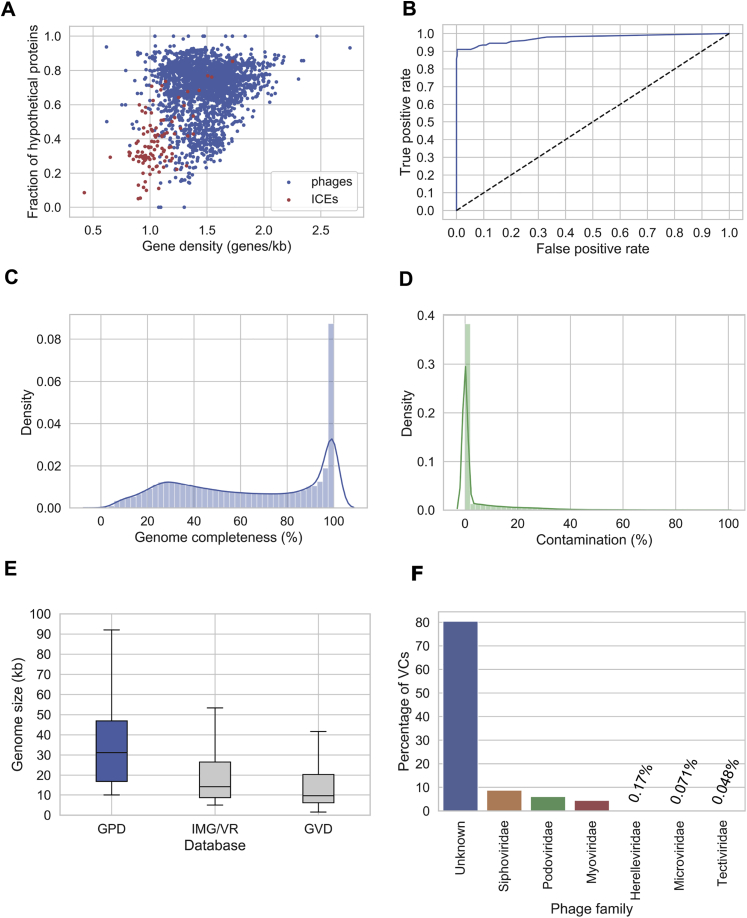
Generating the most complete sequence database of human gut bacteriophages, related to Figure 1 **A)** Gene density and fraction of hypothetical proteins are features that can be harnessed discriminate phages from ICEs. **B)** ROC curve showing the high performance (AUC > 0.97) of the neural network developed to decontaminate ICEs from phages. **C**) Genome completeness distribution as estimated by CheckV on GPD. **D)** GPD contamination distribution according to CheckV. **E)** Size distribution of GPD against other public databases. **F)** Assignment of viral taxonomy to GPD predictions.

To further improve the quality of the dataset, we devised a machine-learning approach to filter out contaminant mobile genetic elements (MGEs) (Figure S1A). We identified predictions carrying machinery from type IV secretion systems, suggesting contamination by conjugative mobile elements, such as plasmids or integrative and conjugative elements (ICEs). We used a feedforward neural network to discriminate phages from ICEs by exploiting differences in gene density, fraction of hypothetical proteins, and k-mer composition signatures (see STAR methods). The classifier was trained with experimental sequences of phages and ICEs and showed an excellent performance in an independent test set (area under the curve [AUC] > 0.97) (Figure S1B) of human gut mobile genetic elements. Next, we dereplicated the final set of filtered sequences at a 95% average nucleotide identity (ANI) threshold (over a 75% aligned fraction) obtaining a database of 142,809 gut phage sequences, henceforth referred to as the GPD.

**Figure S2:**
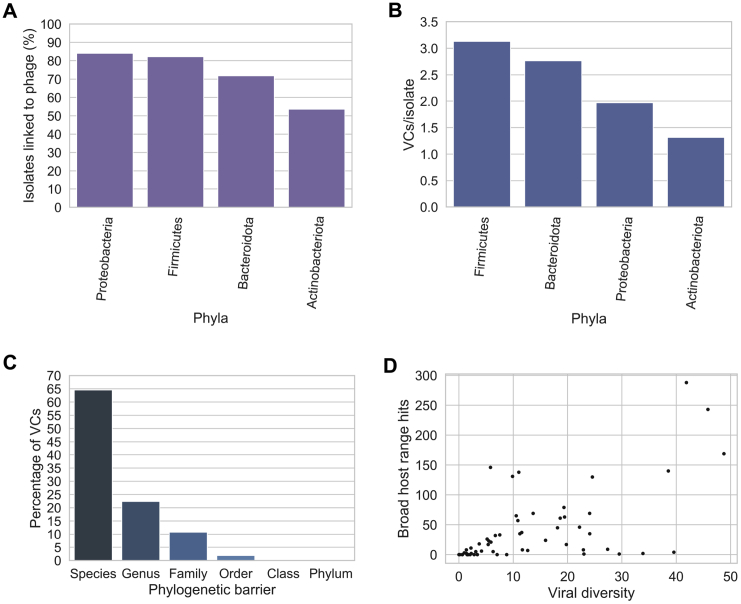
Bacterial host assignment and host range for gut phage, related to Figure 2 **A)** Percentage of isolates of each phylum linked to phage by CRISPR spacers and prophage assignment. Actinobacteria had the lowest percentage of isolates predicted to be a phage host. Actinobacteria versus Bacteroidota (p = 0.007, test), Actinobacteria versus Proteobacteria (p = 0.0025, test), Actinobacteria versus Firmicutes (p = 1.01 × 10^−5^, test). **B)** The Firmicutes hosted the highest viral diversity (highest number of VCs/isolate). Firmicutes versus Bacteroidota (p = 0.021, test), Firmicutes versus Proteobacteria (p = 4.41 × 10^-6,^ test), Firmicutes versus Actinobacteriota (p = 1.1 × 10^−31^, test) **C)** The majority of VCs were found to be restricted to infect a single species. However, a considerable number of VCs (~36%) had a broader host range (p = 0.0, binomial test). **D)** In general, the higher the viral diversity per bacterial genus, the higher the number of phages with broad host range (Spearman’s Rho = 0.6685, p = 3.91x10^−9^).

We estimated the level of completeness of each viral genome with CheckV (Nayfach et al., 2020) (Figure 1B). This tool infers the expected genome length of a viral prediction based on the average amino acid identity to a database of complete viral genomes from NCBI and environmental samples. In total, 13,429 (9.4%) of the viral genomes were classified as complete, 27,999 (19.6%) as high quality, and 101,381 (70.99%) as genome fragments (<90% complete). This classification scheme is consistent with the Minimum Information about an Uncultivated Virus Genome standards (Roux et al., 2019). The median genome completeness of all genomes stored in the GPD was estimated to be 63.5% (interquartile range, IQR = 34.68%–95.31%) (Figure S1C). Estimation of non-viral DNA by CheckV showed that 73.5% of GPD predictions had no contamination whereas 84.13% had a predicted contamination <10% (Figure S2D). In comparison to other human gut phage databases (Gregory et al., 2019; Paez-Espino et al., 2019), GPD had the largest median genome size with ∼31 kb, followed by IMG/VR and GVD with 15 kb and 11 kb, respectively (Figure S1E).

### GPD significantly expands gut bacteriophage diversity

In order to assess the viral diversity of the GPD at high taxonomic levels, we used a graph-based clustering approach to group genetically related phages. Merging GPD with the RefSeq phages and 2 other human gut phage databases (GVD and gut phage fraction of IMG/VR) resulted in the generation of 21,012 non-singleton viral clusters (VCs) with at least 1 GPD prediction (GPD VCs). A VC corresponds to a viral population sharing approximately 90% sequence identity over ∼75% aligned fraction (see STAR methods for further details). Benchmarking against the RefSeq phages (Brister et al., 2015) revealed that the boundaries of GPD VCs were equivalent to a subgenus level, as 99.73% of all VCs were contained within the genus level.

Strikingly, less than 1% (171 out of 21,012) of the GPD VCs overlap with the RefSeq phages. Phages from these 171 VCs mainly infect *Escherichia*, *Enterobacter*, *Staphylococcus*, and *Klebsiella* genera, reflecting the bias of the RefSeq database toward well-known clinically important and traditionally cultured bacteria. Consistent with previous reports of phage predictions from metagenomic datasets (Hoyles et al., 2014), we were not able to confidently assign a family to the majority (∼80%) of GPD VCs, whereas the rest corresponded mainly to the *Podoviridae*, *Siphoviridae*, and *Myoviridae* families (Figure S1E). These 3 viral families belong to the *Caudovirales* order (phages characterized by having tails and icosahedral capsids) and were previously reported to be enriched in human feces (Hoyles et al., 2014; Roux et al., 2012).

For comparison purposes, we also considered VCs without GPD predictions (Figure 1C). Analysis of VCs composed from only GPD and IMG/VR genomes showed 3,699 overlaps, whereas we found 3,206 VCs composed of only GPD and GVD genomes. Moreover, GPD harbored the highest number of unique VCs with 12,731 clusters. On the other hand, 1,099 VCs and 113 VCs were unique to IMG/VR and GVD, respectively. In addition, 1,205 VCs were shared by the 3 databases. Interestingly, the number of VCs with an assigned phage taxon was lower in the VCs that were unique to GPD as opposed to those shared with GVD and IMG/VR (18.74% versus 27.8%) (p = 1.96 × 10^−9^, χ2 test). Thus, GPD considerably expanded the previously unknown gut phage diversity in the human gut. This phage diversity expansion is likely driven by the high number of gut metagenomes mined and their global distribution, which allows the retrieval of rarer gut phage clades.

In addition, GPD provides an unparalleled opportunity to explore the functions encoded by human gut phages. After clustering the whole proteome of GPD into 202,192 protein clusters, we found that top functions corresponded to DNA binding proteins, integrases, methylases, peptidases, and tape measure proteins; however, the majority of phage proteins (47.46%) could not be assigned a function.

### Bacterial host assignment and host range for gut phage

The GPD creates a unique opportunity to assign specific phage to bacterial host species providing a phylogenetic framework to study gut bacteria-phage biology. Accordingly, we inferred the most likely bacterial hosts for each phage prediction with a comprehensive collection of 2,898 high-quality human gut bacterial isolate genomes (Forster et al., 2019; Zou et al., 2019). By screening for the presence of CRISPR spacers targeting phages and by linking the prophages to their assemblies of origin (Edwards et al., 2016), we assigned 40,932 GPD phages (28.66% of all predictions) to 2,157 host strains. This corresponded to at least 1 phage for 74.43% of all cultured human gut bacteria. In addition, co-occurrence analysis between a phage and its predicted genera host revealed that they were found in the same metagenomic sample 92% of the time. We then analyzed whether there was any preference for phage infection across 4 common human gut bacterial phyla (Firmicutes, Bacteroidetes, Proteobacteria, and Actinobacteriota) (Figure S2A). At the phylum level, we detected significant lower phage prevalence in Actinobacteriota, with 58.79% infected isolates in comparison with at least 70% for the other phyla.

We then measured viral diversity (measured by the number of VCs per isolate) within each phylum. This analysis revealed that the Firmicutes harbor a significantly higher viral diversity (Figure S2B), with an average of 3.13 VCs per isolate while also harboring 60% of the total VCs assigned across all phyla. Interestingly, the Firmicutes diversity was unevenly distributed as most of the viral diversity originated from the Negativicutes and Clostridia classes, with an average of 4.88 VCs and 3.9 VCs per isolate, respectively, in contrast with the Bacilli (0.99 VC per isolate), and none for Bacilli A and Desulfitobacteriia classes.

**Figure 2:**
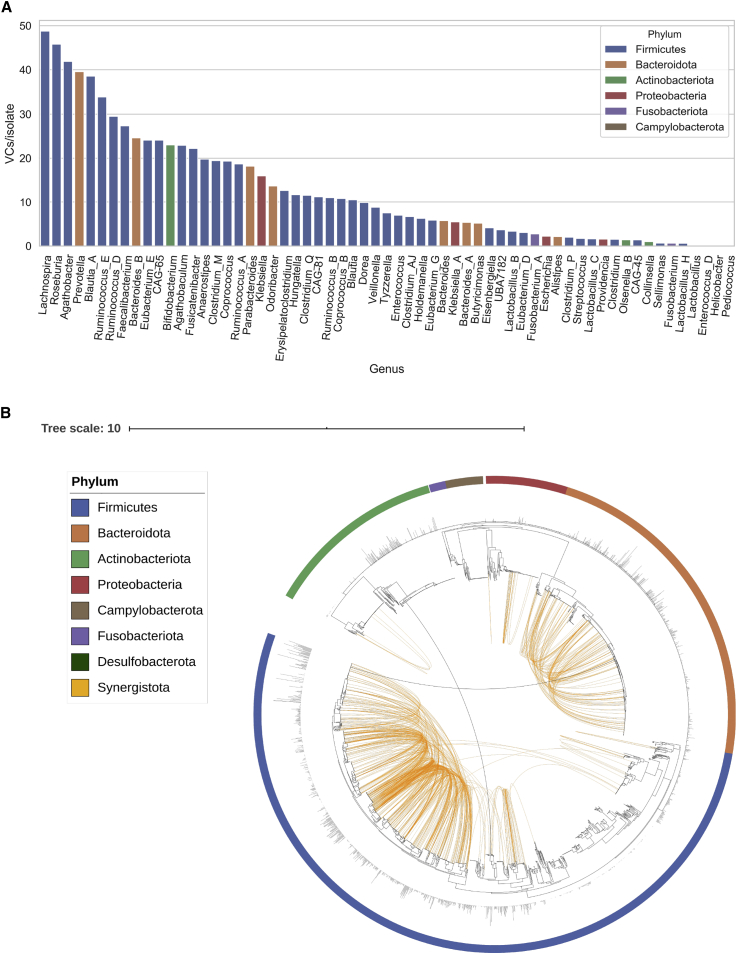
Bacterial host assignment and host range for gut phage (A) Bacterial genera with the highest viral diversity were *Lachnospira*, *Roseburia*, *Agathobacter*, *Prevotella*, and *Blautia* A. On the other hand, the lowest viral diversity was harbored by *Helicobacter* and the lactic acid bacteria *Lactobacillus*, Lactobacillus H, *Enterococcus* D, and *Pediococcus*. (B) Phylogenetic tree of 2,898 gut bacteria isolates showing phage host range. Host assignment was carried out by linking prophages with their assemblies and CRISPR spacer matching. Orange connections represent VCs with a very broad host range (not restricted to a single genus). Black connections represent VCs able to infect 2 phyla. Outer bars show phage diversity (VCs/isolate).

Analysis at the bacterial genus level across all phyla revealed that *Lachnospira*, *Roseburia*, *Agathobacter*, *Prevotella*, and *Blautia* A contain the highest number of VCs per isolate (Figure 2A). With the exception of *Prevotella*, which belongs to the Gram-negative Prevotellaceae family, these genera are members of the Gram-positive Lachnospiraceae family of Firmicutes associated with butyrate-producing spore-formers. In contrast, the lowest viral diversity per isolate was detected among *Helicobacter*, and the lactic acid bacteria *Lactobacillus* H, *Lactobacillus*, *Enterococcus* D, and *Pediococcus.* Thus, we observe a wide distribution of phage abundance and prevalence across human gut bacteria, even within the same phylum.

Horizontal transfer of genes between bacteria via transduction is a major driver of gene flow in bacterial communities (Chen et al., 2018). Host tropism of bacteriophage is believed to be limited by phylogenetic barriers, with most phages being usually restricted to a single host bacterial species (Ackermann, 1998). However, this has not been investigated at large scale across the human gut bacteria. Host assignment at different bacterial taxonomic ranks revealed that the majority of VCs were restricted to infect a single species (64.51%) (Figure S2C). We also found many VCs with broader host ranges such as those restricted to a single genus (22.39%), family (10.79%), order (1.86%), class (0.26%), and phylum (0.13%). Our findings are in line with a recent survey of the host range of gut phages by meta3C proximity ligation (6,651 unique host-phage pairs) which found that ∼69% of gut phages were restricted to a single species (Marbouty et al., 2020). Visualization of very broad range VCs (i.e., those not restricted to a single genus) reveals the large-scale connectivity between phylogenetically distinct bacterial species that fuels bacteria adaptation and evolution (Figure 2B). In general, the higher the viral diversity per bacterial genus, the higher the number of phages with broad host range (Figure S2D).

Surprisingly, 2 VCs (VC_269 and VC_644) had a host range that spanned 2 bacterial phyla. VC_269 was predicted to infect *Faecalibacterium prausnitzii* C (Firmicutes) and 2 *Bifidobacterium* spp. (Actinobacteriota), whereas VC_644 had a host range that included 5 *Bacteroides* spp. (Bacteroidota) and *Blautia* A *wexlerae* (Firmicutes). We predicted VC_269 to be a *Myoviridae* phage; on the other hand, we could not assign a taxonomy rank to VC_644. The presence of integrases in both VCs suggest that these are temperate phages. We hypothesize that additional phages infecting both Actinobacteriota and Firmicutes might be more common, because recent evidence supports a shared ancestry between phages that infect both Actinobacteria (*Streptomyces*) and Firmicutes (*Faecalibacterium*) (Koert et al., 2019).

Taken together, we reveal that approximately one third of gut phage have a broad host range not limited to a single host species. Our analysis provides a comprehensive blueprint of phage-mediated gene flow networks in human gut microbiome.

### Human lifestyle associated with global gut distribution of phageome types

**Figure S3:**
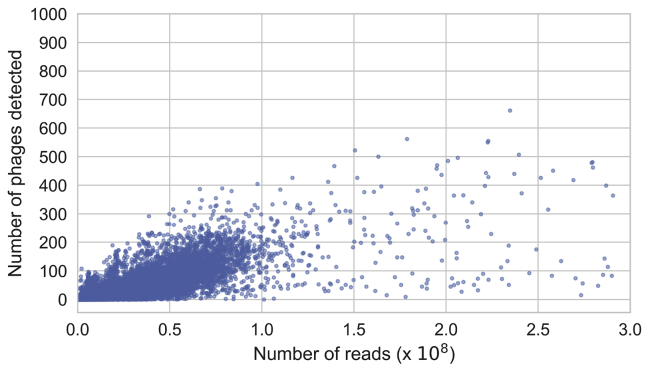
Relationship between sample sequencing depth and phage richness, related to Figure 3 Samples exhibit a positive correlation between sequencing depth and number of phage genomes detected. In order to reduce this bias, we analyzed only samples with a sequencing depth > 50 million reads/sample. Correlation of samples with sequencing depth < 50 million (Pearson’s r: 0.6825, p = 0.0). Correlation of samples with sequencing depth > 50 million (Pearson’s r: 0.3681, p = 2.79x10^−97^).

The gut phageome can be defined as the aggregate of phages that inhabit the gut (Manrique et al., 2016). We performed the most comprehensive phageome profiling of the human gut by read mapping 28,060 metagenomes against the GPD. These metagenomic datasets used to generate the GPD were sampled from 28 different countries across the 6 major continents (Africa, Asia, Europe, North America, South America, and Oceania). Our initial analysis demonstrated a positive correlation between sample sequencing depth and the number of viral genomes detected for samples with <50 million reads. Therefore, we focused further analysis on a dataset of 3,011 deeply sequenced (> 50 million reads) metagenome samples spanning all continents and 23 countries (Figure S3A).

**Figure 3:**
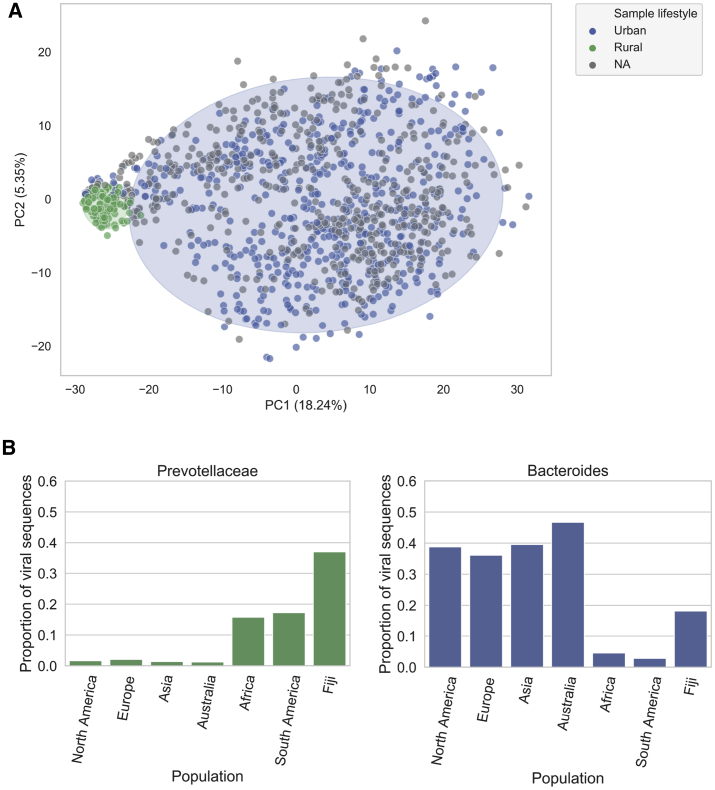
Global phylogeography of gut phages (A) Principal-component analysis (PCA) plot of inter-sample Jaccard distance. Lifestyle is associated with differences in the gut phageome across human populations. Samples from Peru, Madagascar, Tanzania, and Fiji are found in the rural cluster, whereas those samples with a more Westernized lifestyle (mainly from North America, Europe, and Asia) are found in the urban cluster (p = 0.001, R^2^ = 0.36, PERMANOVA test). Ellipses enclose samples within 2 standard deviations for each lifestyle. (B) The proportion of viral sequences (at 95% sequence identity dereplicated) that target Prevotellaceae hosts in traditional societies is higher than that of industrialized populations. Conversely, Bacteroides hosts are more common in industrialized populations than in traditional societies. This result suggests that the composition of the gut phageome at a global scale is driven by the bacterial composition.

We observed clear separation of the North American, European, and Asian phageomes from African and South American samples when we computed the inter-sample Jaccard distance (Figure 3A) (p = 0.001, PERMANOVA test). Interestingly, these phageome patterns are associated with important differences in human lifestyles. Country-wise, samples derived from Africa and South America were mainly sampled from Peru, Tanzania, and Madagascar. Specifically, Peruvian and Tanzanian samples originate from hunter-gatherer communities whereas Malagasy samples come from rural communities with non-Western lifestyles. Oceania was a special case because it had a similar fraction of samples belonging to both groups. However, when we stratified by country, we revealed that all Fijian samples clustered with the rural group, whereas Australian samples segregated with the urbanized cluster. Fiji samples were derived from rural agrarian communities. These observations support the hypothesis that lifestyle, particularly urbanization, could drive differences in the gut phageomes across different human populations.

We reasoned that the bacterial composition of an individual’s microbiome would shape the gut phageome. Prevotellaceae bacteria are more abundant and prevalent in individuals living a rural/traditional lifestyle, whereas *Bacteroides* are more abundant and prevalent in individuals living an urban/western lifestyle (Wu et al., 2011). By harnessing the host assignment data for each phage, we found a significantly higher proportion of VCs assigned to the Prevotellaceae family from African, South American, and Fijian metagenome samples than to those of North America, Europe, Asia, and Australia (p = 0.0, χ2 test) (Figure 3B). Conversely, the *Bacteroides* phages were significantly more prevalent in North America, Europe, Asia, and Australia gut microbiomes (p = 1.72 × 10^−208^, χ2 test). Given the correlation between microbiome enterotypes and phageome types, driven by the intimate connection between phages and their bacterial hosts, we provide evidence that human lifestyle is associated with global variation of gut phageomes, most likely mediated by differences in the host gut microbiome.

### Global distribution of 280 phage VCs

**Figure 4:**
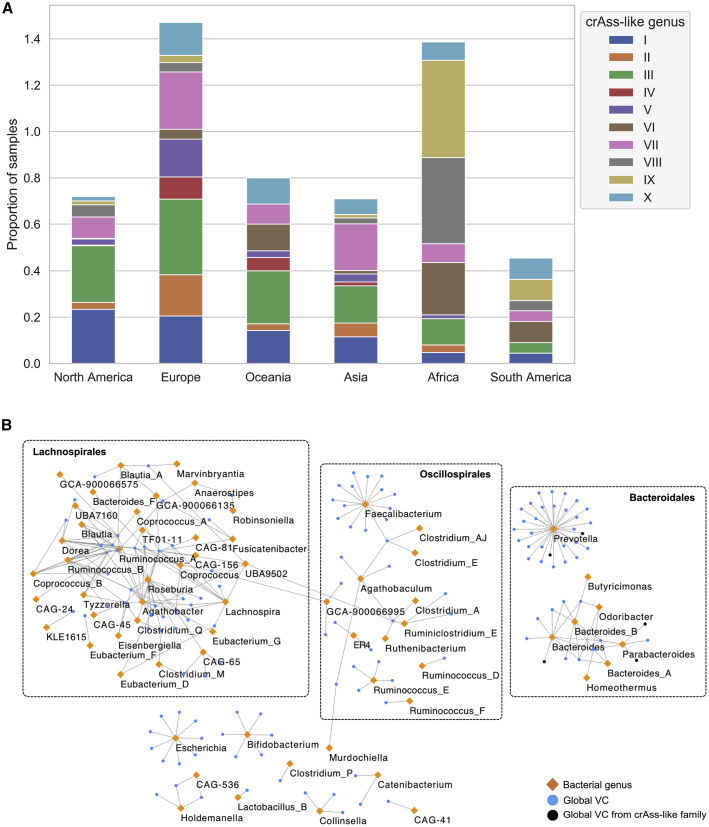
Global gut phage clades and their bacterial hosts (A) The crAss-like family is a globally distributed phage. Genera VI, VIII, and IX—which are predicted to infect a *Prevotella* host—are more common in Africa and South America than are genus I, which infects a *Bacteroides* host. (B) Host-phage network of globally distributed VCs (orange) reveals that *Prevotella*, *Faecalibacterium*, and *Roseburia* are the most targeted bacterial genera. In contrast to the Bacteroidales and Oscillospirales, the VCs from the Lachnospirales are highly shared. VCs that belong to the crAss-like family are highlighted in black; these were predicted to infect *Prevotella*, *Bacteroides*, and *Parabacteroides*.

If the gut phageome is predominantly shaped by the bacterial composition, we would expect to observe strong correlation between the prevalence of VCs with that of their bacterial hosts. A clear example is the crAss-like family of gut phages, which can be divided into 10 phage genera (Guerin et al., 2018). Genus I, which has been found in a large fraction of western microbiome samples is able to infect species from the *Bacteroides* genus. In contrast, genera VIII and IX were previously found to be the most prevalent crAss-like phage among Malawian samples (Guerin et al., 2018). Here, by using CRISPR exact matches, we predict that the most probable host of these 2 phage genera is *Prevotella copri* (rest of crAss-like family predicted hosts in Table S1). In accordance with the results from the Malawian samples, we also found the prevalence of genera VI, VIII, and IX to be higher than that of genus I in Africa, South America, and Fiji (Figure 4A). Thus, the crAss-like family is globally distributed with distinct global distribution patterns at the genera level, which appears to be strongly influenced by human lifestyles and enterotypes.

We further investigated if we could identify other gut phage VCs with global distributions. By extending the analysis to all the VCs, we were able to detect a total of 280 VCs that were globally distributed (found in at least 5 continents). This represents ∼1.3% of all defined VCs (280/21,012).

For 119 out of the 280 VCs (42.5%), we were able to classify them to the *Caudovirales* order, and the remaining 57.5% remained unclassified. When we looked at viral families detected within the *Caudovirales*, we detected *Podoviridae* (10 VCs), *Myoviridae* (28 VCs), *Siphoviridae* (43 VCs), and the newly formed family *Herelleviridae* (1 VC). In addition, when we examined at the phage subfamily level, the most common hits corresponded to the *Picovirinae* and *Peduovirinae* subfamilies with 4 VCs each. Importantly, the genomes of 131 members of 57 globally distributed VCs were mined directly from genomes of cultured isolates, providing unique opportunities for follow-up experiments to study bacteria-phage co-evolution (Table S2).

**Figure S4:**
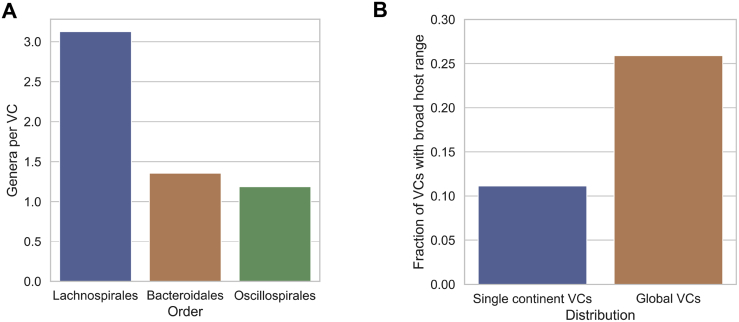
Global gut phage clades and their bacterial hosts, related to Figure 4 **A)** When analyzing globally distributed VCs, the VCs from the order of Lachnospirales were shared across a wider range of genera than those within Oscillospirales and Bacteroidales. Lachnospirales versus Bacteroidales (p = 9.99 × 10^−6^, test). Lachnospirales versus Oscillospirales (p = 6.55 × 10^−6^, test). **B)** We observed that globally distributed phages had a significantly broader range (above genus) than phages found in single continents (p = 1.63 × 10^−5^, test).

A bacteria-phage network of globally distributed VCs (Figure 4B) revealed that *Prevotella* was the most targeted genus (37 VCs), followed by *Faecalibacterium* and *Roseburia* with 15 VCs each. In addition, we observed that in contrast to the Bacteroidales and Oscillospirales, the global VCs associated to the Lachnospirales were highly shared between different genera (Figure S4A). Notably, although 12 globally distributed VCs were members of the crAss-like family (in black), we were only able to assign a host to 6 VCs that targeted Bacteroidales bacteria. We observed that globally distributed phages had a significant broader range (across different genera) than did phages found in single continents (p = 1.62 × 10^−5^) (Figure S4B). This result suggests that broad host-range of certain VCs likely contribute to their expansion across human populations.

Thus, we show that along with 12 crAss-like VCs, there exists a set of at least 280 VCs that is globally distributed. Functional characterization of members of this set will prove useful to shed light on what makes a gut phage to become widespread across human populations.

### The Gubaphage is a highly prevalent clade in the human gut

**Figure 5:**
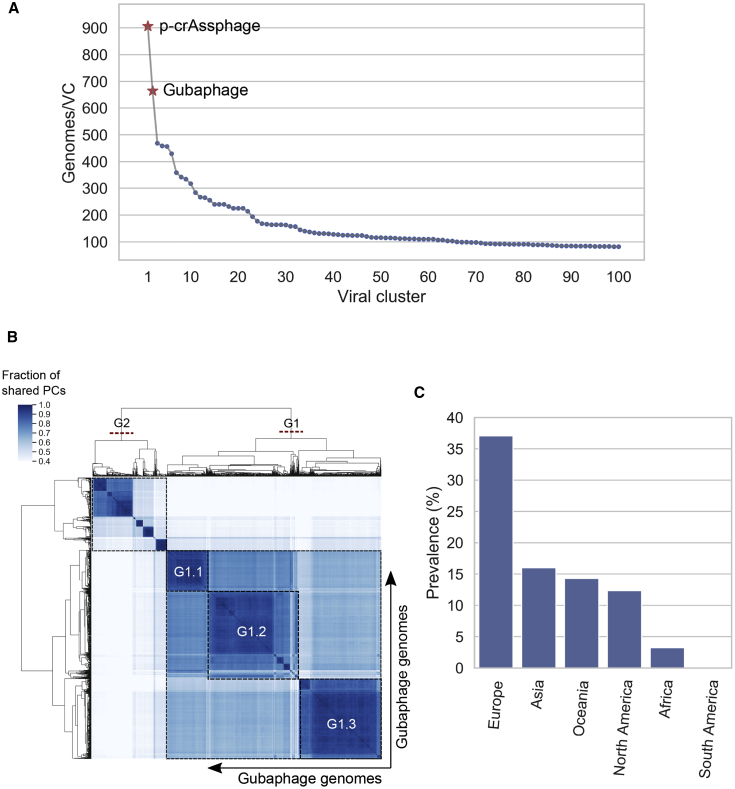
The Gubaphage is a highly prevalent clade in the gut (A) VCs composed of only GPD predictions ranked by number of genomes. VC_3, which belongs to the Gubaphage clade, was the second biggest cluster after VC_1 (composed of p-crAssphage genomes). (B) Analysis of Gubaphage phylogenetic structure revealed 2 genera infecting members of the *Bacteroides* (G1) and *Parabacteroides* (G2). (C) The Gubaphage clade was found in 5 continents, with Europe harboring the highest number of infected samples (38%), as opposed to South America, with none detected.

When we calculated the number of genomes per VC, we discovered that VC_3 had the highest number of GPD predictions, only surpassed by VC_1 (which was composed of p-crAssphage genomes) (Figure 5A). Similar to p-crAssphage, VC_3 was characterized by a long genome (∼80 kb), a Bacteroidetes-Associated Carbohydrate-binding Often N-terminal [BACON] domain-containing protein, and predicted *Bacteroides* host range.

**Figure S5:**
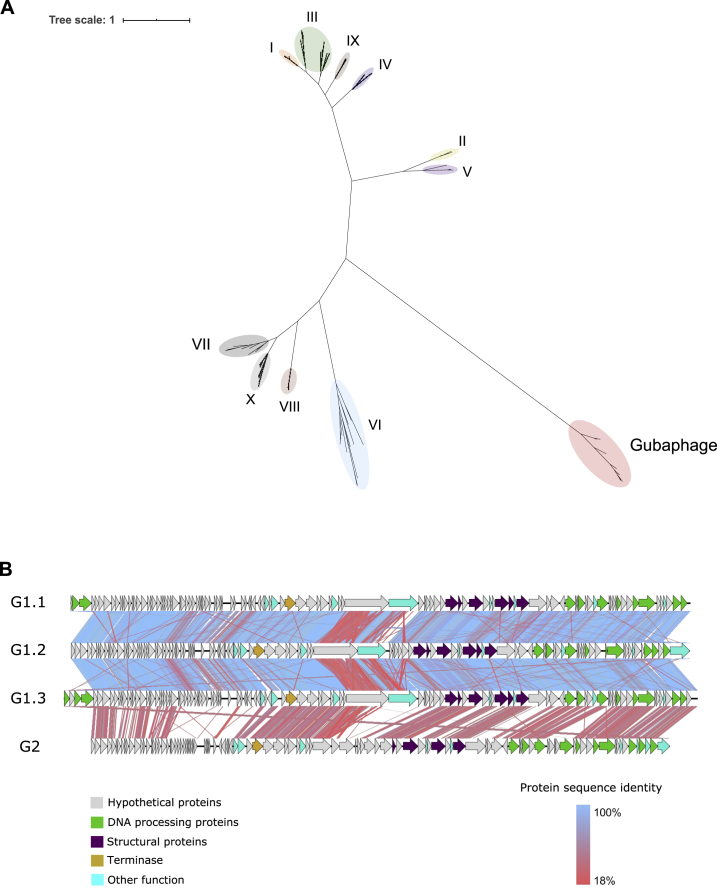
The Gubaphage is a highly prevalent clade in the human gut, related to Figure 5 **A)** Unrooted phylogenetic tree of the large terminase gene from 226 crAss-like genomes and 44 Gubaphage sequences with complete (non-truncated) terminases. Roman numerals correspond to the 10 crass-like genera. The Gubaphage significantly diverged from other crAss-like phages forming a distant clade of its own (red). **B)** Genome wide comparison across Gubaphage clades. The three main regions in which the Gubaphage genome is divided can be appreciated (segment with a run of hypothetical proteins, DNA processing and structural proteins). There is a high protein sequence similarity among members of the G1 clade compared to those of G2.

We identified other 205 VCs by searching for sequences in GPD with significant similarity to VC_3 large terminase gene (E-value < 1 × 10^−6^). We refer to this clade of phages as the Gut Bacteroidales phage (Gubaphage). Given its reminiscent features to crAssphage, we decided to investigate whether the Gubaphage belonged to the recently proposed crAss-like family, which consists of 10 genera and 4 subfamilies (Guerin et al., 2018). We examined this relationship by building a phylogenetic tree using the large terminase gene (Figure S5A). The tree successfully clustered all the crAss-like genera as expected; however, the Gubaphage significantly diverged from the other crAss-like phages, forming a distinct clade.

Given the large genetic diversity contained in Gubaphage’s VC, we sought to characterize its phylogenetic structure (Figure 5B). Analysis of protein overlap between Gubaphage’s genomes revealed that this clade is composed of 2 clusters that share more than 20% but less than 40% of homologous proteins between them. This structure suggests 2 genera (G1 and G2) from a single viral subfamily. In addition, within G1 we identified another phylogenetic substructure composed of 3 large clusters (G1.1, G1.2, and G1.3) composed of 313, 514, and 502 phage genomes, respectively. Host range prediction revealed that G1.1 infects *Bacteroides caccae* and *Bacteroides xylanisolvens* B, G1.3 *Bacteroides* B *vulgatus*, and G2 *Parabacteroides merdae* and *Parabacteroides distasonis*. In the case of G1.2 we couldn’t confidently predict a putative host. Interestingly, the larger genetic distance between G1 and G2 also resulted in a more extreme host range switch, from Bacteroidaceae (G1) to Porphyromonadaceae (G2). Core genes of the Gubaphage included homing endonucleases, DNA polymerase I, FluMu terminase, DNA primase, DNA helicase, Thymidylate kinase, and dUTPase, among others. Annotation of its genome revealed that Gubaphage is organized into 3 distinct regions (Figure S5B). One region encodes structural proteins, the second is composed mainly of genes involved in DNA processing, and the third codes for a series of hypothetical proteins.

Analysis of the distribution of the Gubaphage clade revealed its presence in all the continents except in South America (Figure 5C). Particularly, it reached a prevalence close to 40% in Europe, whereas the lowest corresponded to Africa (3%). The discovery of the Gubaphage clade is yet another example of a highly prevalent phage in the human gut and highlights the need to perform further culturing and mechanistic studies to better understand its role in the human gut microbiota.

Although individual Gubaphage sequences can be found in public databases, here we bring together a multitude of apparently unrelated gut phage sequences and unify them into a formally defined clade. For instance, the phage FAKO05_000032F (Suzuki et al., 2019), had high sequence identity (>90%) with several members of G1.3. Accordingly, we also highlight the importance of metagenomics to consolidate viruses into specific groups such as the case of the crAss-like family (Koonin and Yutin, 2020).

## Discussion

In this study, we generated and analyzed a collection of ∼142,000 high-quality and non-redundant gut phage genomes recovered from 28,060 worldwide distributed human gut metagenomes and 2,898 gut isolate genomes. To our knowledge, this set represents the most comprehensive and complete collection of human gut phage genomes to date and is complemented by other published gut phage databases. We also recognize that due to the type of metagenomes considered (DNA) and limitations of the phage prediction tools, GPD did not capture the whole diversity of human gut phages, such as those belonging to RNA phages. Importantly, this work shows that it is possible to recover high-quality phage genomes from shotgun metagenomes without the need to enrich for viral-like particles (VLPs) from stool samples prior to sequencing. With our approach, we not only recovered non-integrative phages but also uncovered prophage sequences that might rarely enter the lytic cycle and form VLPs. Because shotgun metagenomes are far more readily available than VLP metagenomes, we had access to an unparalleled number of datasets that enabled us to obtain more complete genomes and viral diversity. The usefulness of mining metagenomes for viral characterization/discovery processes has also been reported by the authors of GVD (Gregory et al., 2020). Our pipeline highlighted the need for stringent quality-control procedures in order to filter out contamination when dealing with predictions of mobile genetic elements such as phages. This is particularly true when mining large-scale datasets, due to the impossibility of manually curating every prediction. As the field moves toward the analysis of larger datasets, we believe that machine-learning approaches (such as the classifier developed in this work) can be harnessed to help mitigate contamination and significantly boost the quality of the final set of predictions.

Grouping our predictions into VCs was a critical aspect to organize and manage the vast number of predictions in our database. VCs allowed us to discover important phageome patterns such as uncovering highly genetically diverse phage clades (p-crAssphage and Gubaphage), inferring host range, evaluating prevalence around the world, and exposing epidemiology associations by profiling the phageome composition of human samples. Although vContact (Bin Jang et al., 2019) has been extensively used to group phage sequences into clusters that roughly correspond to genus level, it was not computationally feasible to use it with our massive database. We foresee that as genomic and phenotypic features of these VCs are studied further, it will be possible to classify them into at least 1 of the 15 hierarchical ranks recommended by the ICTV.

Here we also carried out the most comprehensive analysis of the host range of human gut phages. Although the majority of VCs were found to be restricted to a single bacterial species, a significant percentage (∼36%) was predicted to infect multiple species, genera, families, orders, and even classes. A consequence of broad host range phages is an increased connectivity for horizontal gene transfer events due to transduction, which can result in the creation of gene flow networks between phylogenetically distinct bacterial species in the human gut.

The use of GPD also enabled us to gain insights into the epidemiology of gut phages. Notably, we were able to harness global variation in phage composition to learn that the human gut phageome is associated with the lifestyle of individuals and populations. We showed that phages found in urban samples targeted *Bacteroides* over Prevotellaceae bacteria, whereas rural samples from Peru, Tanzania, Madagascar, and Fiji harbored phages with a host range that targeted Prevotellaceae over *Bacteroides* bacteria. This is yet another result that highlights the importance of the size and diversity of our initial dataset, as we were able to capture the genomes of phages from several understudied regions.

In this work, we also show how the GPD can be harnessed for characterization of other important viral subfamilies from the gut. In particular, we discovered that the Gubaphage clade was actually composed of 2 genera and was able to infect bacteria from the Bacteroidaceae and Porphyromonadaceae families. The combined prevalence of the 2 Gubaphage genera reached a sample proportion between 10%–15% in North America, Oceania, and Asia, whereas in Europe it was found to be infecting bacteria in ∼37% of the samples. These results highlight the importance of establishing well-defined viral gut subfamilies, because the combined effect size of highly related phage genomes could help uncover associations of specific clades with their bacterial hosts and human health.

Having a comprehensive database of high-quality phage genomes paves the way for a multitude of analyses of the human gut virome at a greatly improved resolution, enabling the association of specific viral clades with distinct microbiome phenotypes. Importantly, GPD provides a blueprint to guide functional and phenotypic experiments of the human gut phageome, as we linked over 40,000 predictions to 472 cultured gut bacteria species. GPD also harbors 2,496 phages that were mined from cultured isolates that are publicly available and notably 131 members of 57 globally distributed VCs, providing a resource for wet lab experiments to study bacteria-phage co-evolution. In addition, having more complete genomes allows inspection of the most amenable phages for genetic engineering (Chen et al., 2017) or identification of the receptor-binding protein genes to expand their host range (Yehl et al., 2019). Given how important the mobilome can be for bacterial ecosystems, we believe that further characterization of other prominent genetic elements such as ICEs, integrative mobilizable elements (IMEs), genetic islands, and transposons will bring us closer to understanding the association of the gut microbiome with different lifestyles, age, and, ultimately, health and disease.

## STAR★Methods

### Key Resources Table

**Table:** 

REAGENT or RESOURCE	SOURCE	IDENTIFIER
**Deposited Data**		

GPD database and metadata	This paper	http://ftp.ebi.ac.uk/pub/databases/metagenomics/genome_sets/gut_phage_database/
Classifier scripts	This paper	https://github.com/cai91/GPD/tree/master/classifier

**Software and Algorithms**		

SPAdes v.3.10.0	Bankevich et al., 2012	https://github.com/ablab/spades
MEGAHIT v1.1.3	Li et al., 2015	https://github.com/voutcn/megahit
VirSorter v1.0.5	Roux et al., 2015	https://github.com/simroux/VirSorter
VirFinder v1.1	Ren et al., 2017	https://github.com/jessieren/VirFinder
BLAST v2.6.0	Altschul et al., 1990	+ftp://ftp.ncbi.nlm.nih.gov/blast/executables/blast+
CD-HIT v4.7	Li and Godzik, 2006	https://github.com/weizhongli/cdhit/wiki
TensorFlow v1.10.0	Abadi et al., 2016	https://www.tensorflow.org/install
Keras v2.2.4	–	https://keras.io/
checkV v0.7.0	Nayfach et al., 2020	https://bitbucket.org/berkeleylab/checkv/src/master/
MCL v14-137	van Dongen, 2000	https://www.micans.org/mcl/index.html?sec_software
HMMER v3.1b2	Eddy, 1998	http://hmmer.org/
CrisprCasFinder-2.0.2	Couvin et al., 2018	https://github.com/dcouvin/CRISPRCasFinder
GTDB-Tk v0.3.1	Chaumeil et al., 2019	https://github.com/Ecogenomics/GTDBTk
Prodigal v2.6.3	Hyatt et al., 2010	https://github.com/hyattpd/Prodigal
eggnog-mapper v2.0	Huerta-Cepas et al., 2017, 2019	https://github.com/eggnogdb/eggnog-mapper
Prokka v1.5-135	Seemann, 2014	https://github.com/tseemann/prokka
BWA-MEM v0.7.16a-r1181	Li and Durbin, 2009	https://github.com/lh3/bwa
Kraken2	Wood et al., 2019	https://github.com/DerrickWood/kraken2
MAFFT v7.453	Katoh et al., 2002	https://mafft.cbrc.jp/alignment/software/
Easyfig v2.2.5	Sullivan et al., 2011	https://mjsull.github.io/Easyfig/files.html

**Other**		

ICEberg 2.0	Bi et al., 2012	https://db-mml.sjtu.edu.cn/ICEberg/
ImmeDB	Jiang et al., 2019	http://immedb.org/
GVD	Gregory et al., 2019	https://de.cyverse.org/dl/d/E83EFBFF-2A23-4794-8819-ADD34160D018/FINAL_Gut_Viral_Database_GVD_1.7.2018.fna
IMG/VR	Paez-Espino et al., 2019	https://img.jgi.doe.gov/vr/
ViPhOG v1	This paper	ftp://ftp.ebi.ac.uk/pub/databases/metagenomics/viral-pipeline/hmmer_databases
28,060 human gut metagenomes	European Nucleotide Archive (ENA)	http://ftp.ebi.ac.uk/pub/databases/metagenomics/genome_sets/gut_phage_database/GutMetagenomes_metadata.csv
2,898 gut bacteria isolate genomes	Almeida et al., 2021; Forster et al., 2019; Zou et al., 2019	http://ftp.ebi.ac.uk/pub/databases/metagenomics/mgnify_genomes/

### Resource availability

#### Lead contact

Further information and requests for resources and reagents should be directed to and will be fulfilled by the Lead Contact, Trevor D. Lawley (tl2@sanger.ac.uk).

#### Materials availability

This study did not generate new unique reagents.

#### Data and code availability

GPD sequences and associated metadata can be found in the following FTP link: http://ftp.ebi.ac.uk/pub/databases/metagenomics/genome_sets/gut_phage_database/

Classifier and scripts used to generate figures can be found here:

https://github.com/cai91/GPD

### Experimental model and subject details

References to the original studies that generated the metagenomic samples analyzed in this work can be found in the following FTP link:

http://ftp.ebi.ac.uk/pub/databases/metagenomics/genome_sets/gut_phage_database/GutMetagenomes_metadata.csv

### Methods Details

#### Metagenome assembly

Sequencing reads from 28,060 human gut metagenomes were obtained from the European Nucleotide Archive (Leinonen et al., 2011) Paired-end reads were assembled using SPAdes v3.10.0 (Bankevich et al., 2012) with option ‘–meta’, while single-end reads were assembled with MEGAHIT v1.1.3 (Li et al., 2015) both with default parameters.

#### Viral sequence prediction

To identify viral sequences among human gut metagenomes, we used virFinder (Ren et al., 2017) which relies on k-mer signatures to discriminate viral from bacterial contigs, and VirSorter (Roux et al., 2015) which exploits sequence similarity to known phage and other viral-like features such as GC skew. While VirFinder is only able to classify whole contigs, VirSorter can also detect prophages and thus classifies viral sequences as ‘free’ or integrated. Since obtaining high-quality genomes was paramount for our downstream analyses, we used conservative settings for both tools. Metagenome assembled contigs > 10 kb in length were analyzed with VirSorter v1.0.5 and VirFinder v1.1 to detect putative viral sequences. With VirSorter, only predictions classified as category 1, 2, 4 or 5 were considered. In the case of VirFinder, we selected contigs with a score > 0.9 and p < 0.01. In total, we predicted 697,817 phage genomes > 10 kb.

#### Human decontamination

Contigs were further quality-filtered to remove host sequences using a blast-based approach. Briefly, we first used the ‘blastn’ function of BLAST v2.6.0 (Altschul et al., 1990) to query each contig against the human genome GRCh38 using the following parameters: ‘-word_size 28 -best_hit_overhang 0.1 -best_hit_score_edge 0.1 -dust yes -evalue 0.0001 -min_raw_gapped_score 100 -penalty −5 -perc_identity 90 -soft_masking true’. Contigs with positive hits across > 60% total length were excluded. After applying this filtering we recovered 697,796 sequences.

#### Sequence clustering

Dereplication of the filtered contigs was performed with CD-HIT v4.7 (Li and Godzik, 2006) using a global identity threshold of 99% (‘-c 0.99’). This was performed first on contigs obtained within the same ENA study, and afterward among those obtained across studies. This initial dereplication yielded 375,960 sequences. A final set of representative viral sequences was generated by clustering these resulting contigs at a 95% nucleotide identity over a local alignment of 75% of the shortest sequence (options ‘-c 0.95 -G 0 -aS 0.75’). After the latter dereplication we obtained 198,985 sequences.

#### Quality control of GPD predictions

In order to ensure a high-quality of GPD predictions we removed integrative and conjugative elements by using a machine learning approach. Our training set consisted of all experimental ICEs with intact sequence retrieved from ICEberg 2.0 (Bi et al., 2012) and the phage RefSeq genomes from NCBI (Brister et al., 2015). Our test set was downloaded from the Intestinal microbiome mobile elements database (ImmeDB) corresponding to the “ICEs” and “Prophages” datasets (Jiang et al., 2019). By parsing GFF files with custom Python scripts, for each sequence we calculated 3 high-level features, namely number of genes/kb, number of hypothetical proteins/total genes, and 5-kmer relative frequency (4^5^ = 1024 kmers). We used Keras v2.2.4 with the TensorFlow v1.10.0 (Abadi et al., 2016) backend to train a feedforward neural network with an initial hidden layer of size 10 (ReLU activation), followed by another hidden layer of size 5 (ReLU activation) and a final neuron with a sigmoid activation function. We trained the network using the Adam optimizer and the binary cross entropy as the loss function. Model selection was carried out with 5-fold cross-validation. We carried out the classification by allowing a false positive rate of 0.25% with a recall of 91%. This procedure removed 56,094 sequences predicted as ICEs. Finally, we excluded genomes that were predicted to belong to non-phage taxa, such as *Mimiviridae*, *Poxviridae*, and *Marseilleviridae* (82 predictions).

#### Clustering of phages into VCs

We first created a BLAST database (makeblastdb) of all the nucleotide sequences stored in GPD and then carried out all the pairwise comparisons by blasting GPD against itself (we kept hits with E-value ≤ 0.001). Then, for every pairwise comparison, we calculated the coverage by merging the aligned fraction length of the smaller sequence that shared at least 90% sequence similarity. We kept only the results with a coverage > 75%. Finally, we carried out a graph-based clustering by running the Markov Clustering Algorithm (MCL v14-137) (van Dongen, 2000) with an inflation value of 6.0.

#### Viral taxonomic assignment

Viral taxonomic assignment of contigs was performed using a custom database of phylogenetically informative profile HMMs (ViPhOG v1, available here: ftp://ftp.ebi.ac.uk/pub/databases/metagenomics/viral-pipeline/hmmer_databases), where each model is specific to one viral taxon. We used ‘hmmscan’ from HMMER v3.1b2 (Eddy, 1998) to query each protein sequence against the ViPhOG database, setting a full-sequence E-value reporting threshold of 10^−3^ and a per-domain independent E-value threshold of 0.1. Resulting hits were analyzed to predict the most likely and specific taxon for the whole contig based on the following criteria: (i) a minimum of 20% of genes with hits against the ViPhOG database, or at least two genes if the contig had less than 10 total genes; and (ii) among those with hits against the ViPhOG database, a minimum of 60% assigned to the same viral taxon.

#### Host assignment

We predicted CRISPR spacer sequences from the 2898 gut bacteria using CrisprCasFinder-2.0.2 (Couvin et al., 2018). We only used spacers found in CRISPR arrays having evidence levels 3 and 4. We assigned a host to a prediction only if the putative host CRISPR spacer matched perfectly to the phage prediction (100% sequence identity across whole length of CRISPR spacer). We carried out the screen by blasting all the predicted CRISPR spacers against the nucleotide GPD BLAST database using the following custom settings (task: blastn-short, - gapopen 10, -gapextend 2, penalty −1, -word_size 7 m -perc_identity 100). We kept only hits that matched across the whole length of the spacer with a custom script. In addition, prophages were assigned to the bacterial assembly from which they were predicted. Statistical significance of the co-occurrence analysis between a phage and its host was assessed with a binomial test.

#### Taxonomic assignment of bacterial genomes

Bacterial isolate genomes were taxonomically classified with the Genome Taxonomy Database Toolkit (GTDB-Tk) v0.3.1 (Chaumeil et al., 2019) (https://github.com/Ecogenomics/GTDBTk) (database release 04-RS89) using the ‘classify_wf’ function and default parameters. Taxa with an alphabetic suffix represent lineages that are polyphyletic or were subdivided due to taxonomic rank normalization according to the GTDB reference tree. The unsuffixed lineage contains the type strain whereas all other lineages are given alphabetic suffixes, suggesting that their labeling should be revised in due course.

#### GPD proteome analysis and functional annotation

We predicted the whole proteome of GPD with Prodigal v2.6.3 (metagenomic mode) and masked the low-complexity regions with DustMasker. We then created a BLAST database of all the protein sequences and carried out all the pairwise comparisons by blasting the GPD proteome against itself (we kept hits with E-value ≤ 0.001). Then, for every pairwise comparison, we calculated a similarity metric as defined by Chan et al. (Chan et al., 2013). Finally, we ran the Markov Clustering Algorithm (MCL) with an inflation value of 6.0 and removed clusters with only 1 member. Protein function assignment was carried out by eggnog-mapper v2.0 (Huerta-Cepas et al., 2017, 2019). with default parameters. Individual GFF3 files for each GPD genome were generated using Prokka v1.5-135 (Seemann, 2014).

#### Metagenomic read mapping

To estimate the prevalence of each viral species, we mapped metagenomic reads using BWA-MEM v0.7.16a-r1181 (Li and Durbin, 2009) (‘bwa mem -M’) against the GPD database (clustered at 95% nucleotide identity) here generated. Mapped reads were filtered with samtools v1.5 (Li et al., 2009) to remove secondary alignments (‘samtools view -F 256’) and each viral species was considered present in a sample if the mapped reads covered > 75% of the genome length. Prevalence of bacteria genera was estimated with Kraken2 (Wood et al., 2019) by mapping against the Unified Human Gastrointestinal Genome (UHGG) catalog (Almeida et al., 2021) considering a minimum threshold of a relative abundance of 0.1%.

#### Geographical distribution of metagenomic samples

We removed samples with a sequencing depth below 50 million reads/sample, as below this threshold we observed a positive correlation between sample depth and number of viral genomes detected (Figure S3B). This new subset consisted of 3011 samples and spanned all the continents and 23 countries. Similarity between 2 samples was calculated by computing the number of shared VCs divided by the total number of VCs in both samples (Jaccard index). Distribution of samples was visualized with PCA.

#### Phylogenetic analysis of Gubaphage

The phylogenetic tree comparing Gubaphage against crAss-like phages was constructed by aligning the corresponding large terminase genes with MAFFT v7.453 (Katoh et al., 2002) –auto mode, followed by FastTree v2.1.10 (Price et al., 2010). The resultant tree was visualized on iTOL (Letunic and Bork, 2007). We identified the Gubaphage clades by calculating the fraction of shared protein clusters among all the Gubaphage genomes and then carrying out hierarchical clustering with average linkage and Euclidean metric. Genome-wide comparisons between the Gubaphage clades were generated with Easyfig v2.2.5 (Sullivan et al., 2011), using the tBLASTx algorithm with the following parameters: e-value cutoff 0.001 and length filter 30.

### Quantification and Statistical Analysis

Quantification and analysis procedures of viral prediction, host assignment and epidemiology were provided in the relevant main text or in Method Details. All these tests were performed in Python and a p value < 0.05 was considered statistically significant.
