# Supplementary material for: Massive expansion of human gut bacteriophage diversity
Source: Cell. 2021 Feb 18;184(4):1098–1109.e9. doi: 10.1016/j.cell.2021.01.029 (PMC7895897; doi:10.1016/j.cell.2021.01.029)
Supplement: Document S1. Tables S1 and S2 [file mmc1.pdf]

Cell, Volume *184*

Supplemental Information

Massive expansion of human gut bacteriophage diversity

Luis F. Camarillo-Guerrero, Alexandre Almeida, Guillermo Rangel-Pineros, Robert D. Finn, and Trevor D. Lawley

crAss-like genus	Predicted hosts
I	NA
II	NA
III	*Bacteroides*_B *vulgatus*
IV	*Bacteroides xylanisolvens*_B
	Bacteroides caccae
V	*Fusicatenibacter saccharivorans*
	Lachnospira eligens
VI	*Bacteroides xylanisolvens*_B
	Prevotella copri
VII	*Bacteroides thetaiotaomicron*
	*Bacteroides*_B *massiliensis*
	*Bacteroides*_B *dorei*
	Bacteroides caccae
	Bacteroides faecis
	Bacteroides eggerthii
	*Bacteroides xylanisolvens*_B
	*Bacteroides*_B *vulgatus*
	Bacteroides uniformis
VIII	*Prevotella copri*
IX	*Prevotella copri*
X	*Parabacteroides merdae*
	Parabacteroides distasonis

Table S1. Predicted hosts of the crAss-like family

Global_VC	GPD_phage_ID	Identifier	Source	Sample	Culture Collection	Culture Collection Number
VC_2	ivig_4281	ERR2230082	HBC	Bacteroides dorei ERR2230082	WSI	ERR2230082
VC_2	ivig_3443	ERR1022271	HBC	Bacteroides uniformis ERR1022271	WSI	ERR1022271
VC_2	ivig_4319	ERR2230141	HBC	Bacteroides uniformis ERR2230141	WSI	ERR2230141
VC_2	ivig_4298	ERR2230119	HBC	Bacteroides uniformis ERR2230119	WSI	ERR2230119
VC_2	ivig_3716	ERR1022414	HBC	Bacteroides cellulosilyticus ERR1022414	DSMZ	DSM 108229
VC_2	ivig_3679	ERR2221211	HBC	Bacteroides acidifaciens ERR2221211	WSI	ERR2221211
VC_2	ivig_3715	ERR1022413	HBC	Bacteroides thetaiotaomicron ERR1022413	WSI	ERR1022413
VC_2	ivig_4318	ERR2230138	HBC	Bacteroides uniformis ERR2230138	WSI	ERR2230138
VC_71	ivig_3350	ERR2221142	HBC	Bifidobacterium pseudocatenulatum ERR2221142	WSI	ERR2221142
VC_65	ivig_3375	ERR2221151	HBC	Lachnospiraceae nov. ERR2221151	WSI	ERR2221151
VC_94	ivig_3609	ERR1022353	HBC	Lachnospiraceae nov. ERR1022353	WSI	ERR1022353
VC_65	ivig_3761	ERR1022439	HBC	Eisenbergiella tayi ERR1022439	WSI	ERR1022439
VC_16	ivig_3950	ERR1203972	HBC	Lachnospiraceae nov. ERR1203972	WSI	ERR1203972
VC_50	ivig_4334	ERR2230158	HBC	Bifidobacterium longum ERR2230158	WSI	ERR2230158
VC_50	ivig_4156	ERR2221351	HBC	Bifidobacterium longum ERR2221351	NCIMB	NCIMB 15144
VC_55	ivig_3549	ERR1022318	HBC	Eubacterium rectale ERR1022318	WSI	ERR1022318
VC_75	ivig_3398	ERR2221161	HBC	Lachnospiraceae nov. ERR2221161	WSI	ERR2221161
VC_78	ivig_4245	ERR2221398	HBC	Escherichia coli ERR2221398	WSI	ERR2221398
VC_138	ivig_4044	ERR2221275	HBC	Dorea formicigenerans ERR2221275	WSI	ERR2221275
VC_156	ivig_3731	ERR1022423	HBC	Anaerostipes hadrus ERR1022423	WSI	ERR1022423
VC_156	ivig_3760	ERR1022437	HBC	Fusicatenibacter saccharivorans ERR1022437	CCUG & JCM	CCUG 68552 & JCM 31268
VC_168	ivig_3524	ERR1022310	HBC	Ruminococcus gnavus ERR1022310	DSMZ	DSM 108212
VC_168	ivig_4348	ERR171257	HBC	Ruminococcus gnavus ERR171257	WSI	ERR171257
VC_175	ivig_3612	ERR1022356	HBC	Bifidobacterium pseudocatenulatum ERR1022356	WSI	ERR1022356
VC_183	ivig_3767	ERR1022442	HBC	Ruminococcaceae nov. ERR1022442	WSI	ERR1022442

VC_204	ivig_3670	ERR1022391	HBC	Lachnospira nov. ERR1022391	BCCM	LMG 29490
VC_288	ivig_3565	ERR1022327	HBC	Faecalibacterium prausnitzii ERR1022327	WSI	ERR1022327
VC_752	ivig_3547	ERR1022317	HBC	Ruminococcaceae nov. ERR1022317	WSI	ERR1022317
VC_757	ivig_4035	ERR2230078	HBC	Collinsella aerofaciens ERR2230078	CCUG	CCUG 73007
VC_1461	ivig_4025	ERR2230076	HBC	Lachnoclostridium nov. ERR2230076	WSI	ERR2230076

Table S2. Global VCs found in publicly available gut isolates.
